# *HMGA1P7*-pseudogene regulates *H19* and *Igf2* expression by a competitive endogenous RNA mechanism

**DOI:** 10.1038/srep37622

**Published:** 2016-11-22

**Authors:** Marco De Martino, Floriana Forzati, Marianna Marfella, Simona Pellecchia, Claudio Arra, Luigi Terracciano, Alfredo Fusco, Francesco Esposito

**Affiliations:** 1 Istituto di Endocrinologia ed Oncologia Sperimentale del CNR c/o Dipartimento di Medicina Molecolare e Biotecnologie Mediche, Scuola di Medicina e Chirurgia di Napoli, Università degli Studi di Napoli “Federico II”, via Pansini 5, 80131 Naples, Italy; 2 Istituto Nazionale dei Tumori, Fondazione Pascale, via Mariano Semmola, 80131 Naples, Italy; 3 Institute of Pathology, Molecular Pathology Division, University of Basel, Schonbeinstrasse 40, 4003 Basel, Switzerland

## Abstract

Recent studies have revealed that pseudogene transcripts can function as competing endogenous RNAs, and thereby can also contribute to cancer when dysregulated. We have recently identified two pseudogenes, *HMGA1P6* and *HMGA1P7* for the *HMGA1* gene whose overexpression has a critical role in cancer progression. These pseudogenes work as competitive endogenous RNA decoys for *HMGA1* and other cancer related genes suggesting their role in carcinogenesis. Looking for new *HMGA1* pseudogene ceRNAs, we performed RNA sequencing technology on mouse embryonic fibroblasts deriving from transgenic mice overexpressing *HMGA1P7.* Here, we report that *HMGA1P7* mRNA sustains the *H19* and *Igf2* overexpression by acting as miRNA decoy. Lastly, the expression of *HMGA1P7* was significantly correlated with *H19* and *IGF2* levels in human breast cancer thereby suggesting a role for *HMGA1P7* deregulation in this neoplasia.

Pseudogenes are a subclass of long non coding RNA (lncRNA) sharing high sequence identity with protein-coding parental counterparts. As stated by the GENCODE pseudogene annotations (v.17), there are almost 15,000 human pseudogenes^1^. They frequently possess features, such as premature stop codons, deletions/insertions, or frame shift mutations, that impede them to produce functional proteins. There are three groups of pseudogenes: processed, duplicated, and unitary^1^,^2^. Processed pseudogenes do not have introns and are thought to arise from reverse transcription of mRNA followed by reinsertion into the genome^1^,^2^. Duplicated pseudogenes contain introns and sometimes even upstream regulatory elements since they are produced by gene duplication. For each pseudogene belonging to these two classes there is an associated protein-coding gene that is highly similar in sequence^1^,^2^. The last type of pseudogenes are the unitary ones, which take place when protein-coding genes accumulate mutations and lose their coding potential^1^,^2^. Consequently, unitary pseudogenes do not have parental genes. From the time of their discovery in 1977, pseudogenes have commonly been thought as “biologically inconsequential” and non-functional^3^. However, recent studies have unveiled different mechanisms by which pseudogenes control gene expression such as the generation of siRNAs^4^,^5^, competition for RNA-binding proteins or the translation apparatus^6^,^7^,^8^, and engagement of proteins by pseudogene antisense RNAs to corresponding sites in the parental gene to modify chromatin transcription and remodeling^9^,^10^. The latest function identified for pseudogenes is post-transcriptional regulation of mRNA levels by competing for microRNAs (miRNAs). Indeed, processed pseudogenes maintain 5′ and 3′ untranslated region (UTR) sequences of their parental genes^11^. Given that miRNAs inhibit target gene expression by binding to the 3′ UTR, pseudogenes can be targeted by miRNAs that modify the expression of coding genes. Definitely, pseudogene transcripts exert regulatory control of their parental gene expression levels by competing for the same miRNAs^12^.

We have recently characterized two processed pseudogenes, *HMGA1P6* and *HMGA1P7*, for the *HMGA1* gene that codes for the HMGA1a and HMGA1b proteins highly overexpressed in most of the human malignancies^13^,^14^. Moreover, it has been previously demonstrated an association between HMGA1 overexpression and a poor patient survival^13^, and that their overexpression is even required for cell transformation^15^,^16^, and is able to induce benign and malignant neoplasias in mice^13^. *HMGA1P6* and *HMGA1P7* pseudogenes, present only in human genome, have preserved seed matches for miRNAs targeting the *HMGA1* oncogene. *HMGA1* pseudogenes (*HMGA1Ps*) overexpression, working as competitive endogenous RNA (ceRNA), increases HMGA1 protein levels by blocking the suppression of HMGA1 protein synthesis exerted by miRNAs^17^,^18^,^19^,^20^. *HMGA1Ps* have also oncogenic activity by suppressing apoptosis and promoting cell proliferation and migration^17^,^18^,^19^,^20^. Moreover, we have previously show that *HMGA1Ps* are overexpressed in anaplastic thyroid carcinomas but not in the differentiated ones, indicating a critical role of them in cancer progression^17^. Since the *HMGA1Ps* contain several seed sequences for miRNAs, their overexpression derepresses the expression of different cancer-related genes, as already demonstrated for *HMGA2, VEGF, EZH2*^17^,^18^,^19^,^20^. Therefore, the aim of this study has been to find novel ceRNA interactors differentially expressed in *HMGAP7* transgenic mouse embryonic fibroblasts (MEFs) with respect to the wild-type (WT) ones, which do not express HMGA1 pseudogenes, using a RNA sequencing (RNA-seq) approach. By this analysis, we found a set of mRNAs up- or down-regulated in *HMGA1P7* overexpressing MEFs in comparison with WT cells. Among them, we focused our attention on two of the most overexpressed and *HMGA1P7* miRNA-sharing genes: *H19* and *insulin-like growing factor 2 (Igf2*).

*H19* and *IGF2* genes are closely linked, showing highly similar patterns of gene expression, but they are reciprocally imprinted. In fact, *H19* is expressed solely from the maternally inherited chromosome, whereas *IGF2* expression is from the paternal chromosome. In particular, the noncoding *H19* has a critical role in genomic imprinting during cell growth and development^21^. The loss of imprinting results in misexpression of *H19* and was detected in many tumors including hepatocellular^22^, bladder^23^, gastric^24^,^25^ and colon^26^ cancer. *IGF2* codes for a mitogenic growth factor that is active in early development and has a critical role in embryonic and fetal growth^27^. Increased expression of *IGF2* is a common feature of both pediatric and adult malignancies^27^, and mounting evidence implicates *IGF2* as a major factor contributing to oncogenesis^27^,^28^,^29^. Here, we report that *HMGA1P7* mRNA induces the *H19* and *Igf2* overexpression by acting as miRNA decoy.

## Results

### RNA-seq on *HMGA1P7* transgenic MEFs

**Figure 1:**
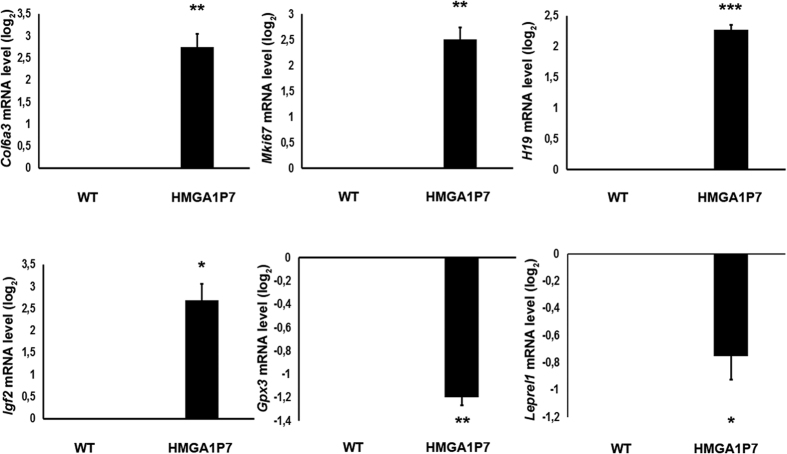
Validation of RNA-seq analyses on *HMGA1P7* MEFs. qRT-PCR analysis of selected deregulated genes from RNA-seq performed on WT and *HMGA1P7* transgenic MEFs. The results are reported as the mean of values. The error bars represent mean ± SE; *P < 0.05 **P < 0.01 ***P < 0.001 (t test).

To identify the genes regulated by *HMGA1P7* expression, we analyzed the whole transcriptome of WT and *HMGA1P7* transgenic MEFs by RNA-seq analyses. To this aim, the entire population of RNA transcripts extracted from WT and *HMGA1P7*-MEFs were sequenced. The genome-wide RNA expression profiles studies reveal that about one hundred fifty transcripts (32 upregulated and 116 downregulated) were regulated by *HMGA1P7* expression with a significant fold-change variation (FDR adjusted p-value of 0.05). To validate the results obtained by RNA-seq, we analyzed the expression of some upregulated genes such as *Collagen Type VI Alpha 3 (Col6a3*), *Marker of Proliferation Ki-67 (Mki67*), *H19, Igf2* and downregulated genes such as *Glutathione Peroxidase 3 (Gpx3*), *Leprecan-Like 1 (Leprel1*) by Real-time PCR (qRT-PCR). As shown in Fig. 1, the quantitative qRT-PCR analyses confirmed the data obtained from the RNA-seq analyses. Interestingly, these genes have been related to several human cancers (colon, gastric, liver, breast and hematological cancers), and are considered possible therapeutic targets^30^,^31^,^32^,^33^,^34^,^35^.

**Figure 2:**
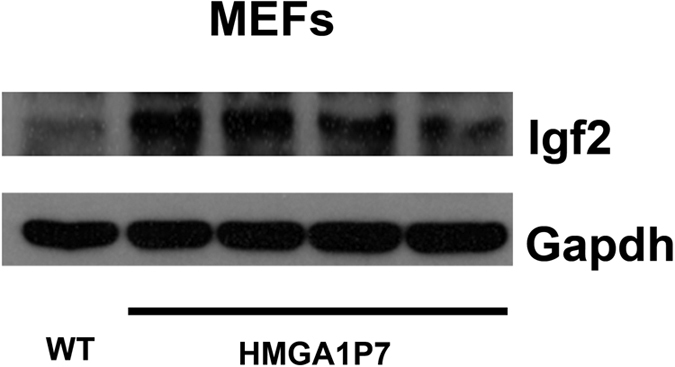
Igf2 is upregulated in *HMGA1P7* MEFs. Western blot analysis of Igf2 from WT and *HMGA1P7* transgenic MEFs.

**Figure 3:**
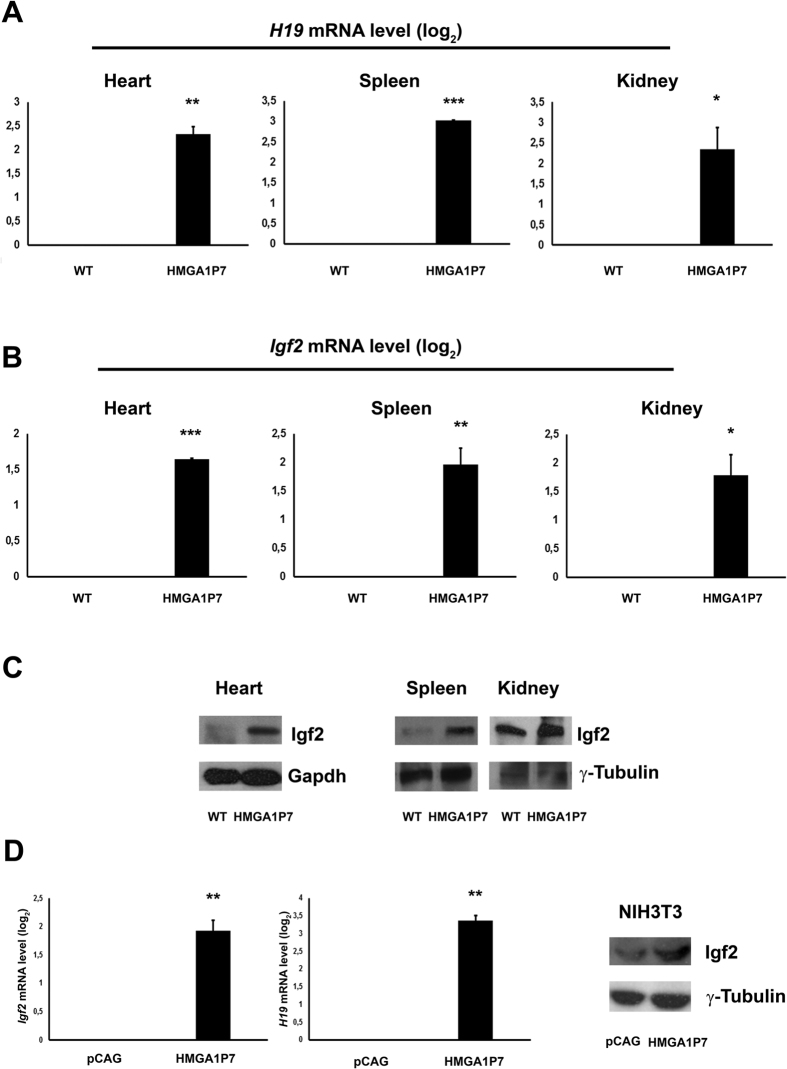
*H19* and *Igf2* are positively regulated by *HMGA1P7.* (**A**) qRT-PCR analysis of *H19* from hearts, spleens and kidneys of WT and *HMGA1P7* transgenic mice. (**B**) qRT-PCR analysis of *Igf2* from hearts, spleens and kidneys of WT and *HMGA1P7* transgenic mice. (**C**) Western blot analysis of Igf2 from heart, spleen and kidney of WT and *HMGA1P7* transgenic mice. (**D**) Left Panel, qRT-PCR analysis of *H19* and *Igf2* from control and *HMGA1P7* overexpressing NIH3T3 cells. Right Panel, Western blot analysis of Igf2 from control and *HMGA1P7* overexpressing NIH3T3 cells. The results are reported as the mean of values. The error bars represent mean ± SE; *P < 0.05 **P < 0.01 ***P < 0.001 (t test).

Among the differentially expressed mRNAs found in MEFs overexpressing *HMGA1P7*, we focused our attention on *H19* and *Igf2* since they, other than to be involved in carcinogenesis, showed the highest fold change among the upregulated genes, and are also targeted by several miRNAs that are able to bind to the *HMGA1P7* mRNA. Western blot analysis for Igf2 confirmed the qRT-PCR data (Fig. 2). Moreover, qRT-PCR and Western blot analysis showed that *H19* and *Igf2* were also upregulated in heart, spleen and kidney from *HMGA1P7* adult transgenic mice (Fig. 3A,B and C). As expected from previous results, qRT-PCR shows upregulation of *H19* and *Igf2* following *HMGA1P7* pseudogene overexpression in NIH3T3 cells (Fig. 3D). Western blot confirms *Igf2* upregulation also at protein level in tissues from *HMGA1P7* and in the *HMGA1P7*-transfected NIH3T3 cells (Fig. 3C and D).

Taken together, these results strongly support the hypothesis that *HMGA1P7* could act as ceRNA for *H19* and *Igf2.*

### *HMGA1P7* act as decoy for *H19* and *Igf2* targeting miRNAs

**Figure 4:**
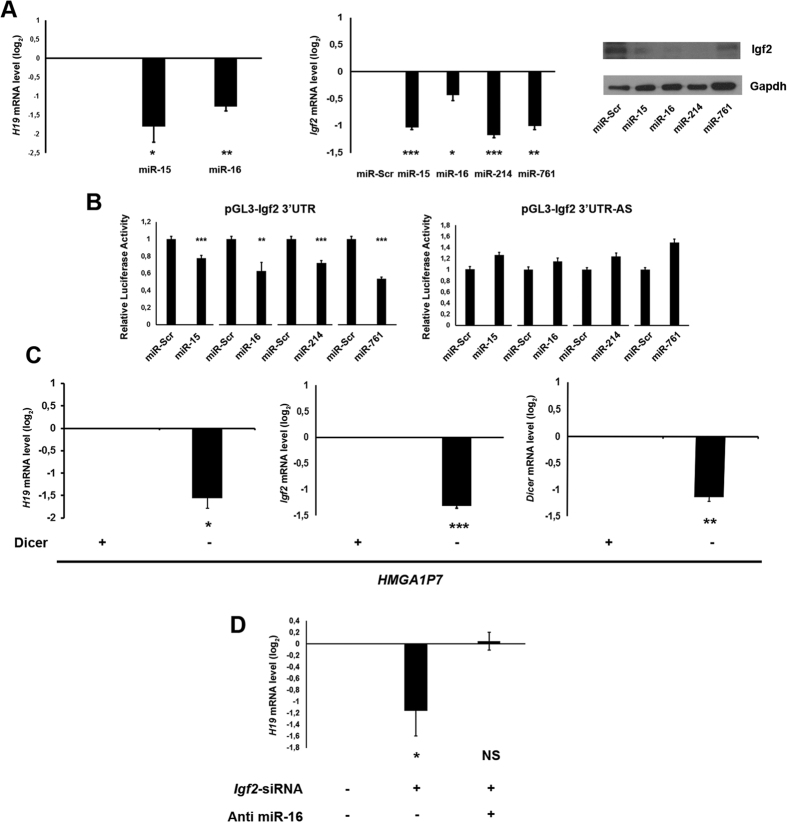
*H19* and *Igf2* are targeted by *HMGA1P7*-targeting miRNAs. (**A**) qRT-PCR analysis of *H19* (left Panel) and *Igf2* (middle Panel) mRNA from the NIH3T3 cells transfected with scrambled-oligonucleotide, miR-15, miR-16, miR-214 and miR-761. Right Panel, Western blot analysis of Igf2 in the same samples as in the middle panel. (**B**) *Igf2* was cloned into the pGL3 control vector. Relative luciferase activity in HEK293 cells transiently transfected with miR-15, miR-16, miR-214, miR-761 and a control scrambled oligonucleotide. (**C**) *H19* and *Igf2* mRNA levels 24 h after the transfection of *HMGA1P7* in scrambled oligonucleotide or siRNA-Dicer NIH3T3 transfected cells. (**D**) qRT-PCR analysis of *H19* mRNA levels from the NIH3T3 cells transfected with siRNA-control, siRNA-*Igf2* alone or in in combination with the Anti miR-16 oligonucleotide.

To test whether the effect of the *HMGA1P7* pseudogene on *H19* and *Igf2* expression is dependent on sharing targeting-miRNAs, we evaluated the ability of *HMGA1P7*-targeting miRNAs^17^ to bind to *H19* and *Igf2*. To this aim, we transfected miR-15, miR-16, miR-214 and miR-761 (already reported to target *HMGA1P7*)^17^ into NIH3T3 cells, and analyzed *H19* and *Igf2* mRNA levels by qRT-PCR. As presented in Fig. 4A, the transfection of the *HMGA1P7*-targeting miRNAs yield a significant reduction of *H19* and *Igf2* mRNA levels. Western blot confirms Igf2 downregulation also at protein level following the transfection of the *HMGA1P7*-targeting miRNAs (Fig. 4A). To define whether the *HMGA1P7*-targeting miRNAs straightly interacted with *Igf2* mRNA, we cloned the *Igf2* 3′ UTR downstream of the luciferase open reading frame. This reporter vector was transfected into NIH3T3 cells together with miRNA precursors and a control non-targeting scrambled oligonucleotide. The luciferase signal was considerably lower after transfection with miR-15, miR-16, miR-214 and miR-761 in comparison with the cells transfected with the scrambled oligonucleotide (Fig. 4B). The overexpression of *H19* and *Igf2* induced by upregulation of *HMGA1P7* was depleted in Dicer-knockdown cells (Fig. 4C) then supporting the hypothesis that *HMGA1P7, H19* and *Igf2* follow the same miRNA-mediated post-transcriptional regulation. In fact, silencing of Dicer, the enzyme that leads miRNA maturation process, results in reduced levels of mature miRNAs compared to control. Moreover, to verify whether *H19* and *Igf2* can act as ceRNA each-other, we transfected siRNA-*Igf2* into NIH3T3 cell line in combination or not with Anti miR-16, which is able to block miR-16 repression on *HMGA1P7, Igf2* and *H19*, and a siRNA-control, then evaluating the *H19* mRNA levels. As proposed by our model, siRNA-*Igf2* transfection induces a significant *H19* downregulation, that is reverted by the transfection with the Anti miR-16 oligonucleotide, suggesting that both *H19* and *Igf2* transcripts can talk each-other through miRNAs mediation (Fig. 4D). These data are consistent with the hypothesis that *HMGA1P7* requires mature miRNAs to regulate *H19* and *Igf2* levels.

### *HMGA1P7, H19* and *IGF2* expression positively correlates in human breast cancer

**Figure 5:**
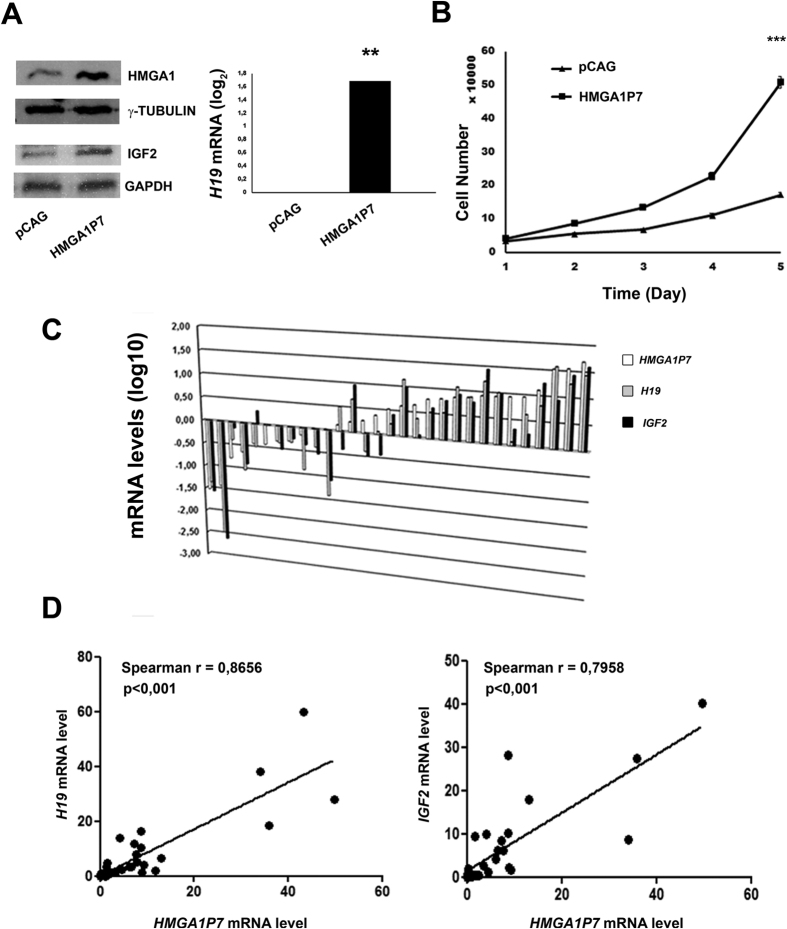
*H19* and *IGF2* expression positively correlates with *HMGA1P7* in breast cancer. (**A**) Left Panel, Western blot analysis of HMGA1 and IGF2 protein levels in control and *HMGA1P7*-overexpressing MCF7 cells. Right Panel, qRT-PCR analysis of *H19* expression of control and *HMGA1P7*-overexpressing MCF7 cells. (**B**) MCF7 cell proliferation of control and *HMGA1P7*-overexpressing cells (**C**) qRT-PCR analysis in tumor and normal breast tissues. The fold change indicates the relative change in expression levels between tumor samples and normal samples, assuming that the value of normal sample is equal to 1. (**B**) Correlation analysis of *HMGA1P7* versus *H19* and *HMGA1P7 versus* *IGF2* are shown. The Spearman’s rank correlation coefficient is shown.

Then, we investigated whether *HMGA1P7* functions as ceRNA through, or partially through *H19,* IGF2 and HMGA1 in breast cancer human cells. As expected, we found upregulation of *H19,* IGF2 and HMGA1 following *HMGA1P7* overexpression in MCF7 cells (human breast adenocarcinoma cell line) (Fig. 5A). Moreover, MCF7-*HMGA1P7* cells grow faster than the control transfected cells as consequence of *HMGA1P7* ceRNA pathway activation (Fig. 5B).

To confirm whether *HMGA1P7* works as miRNA sponge for the regulation of *H19* and *IGF2* expression levels also in human cancer, we evaluated the expression of *H19, IGF2* and *HMGA1P7* in a panel of breast carcinoma samples by qRT-PCR, since *H19* and *IGF2* have been reported to be overexpressed in this type of tumor^36^,^37^. As shown in Fig. 5C, *HMGA1P7* was overexpressed in most of the carcinoma samples as well as *H19* and *IGF2.* Moreover, the direct correlation between *HMGA1P7* and *H19* expression (Spearman r = 0,8656; p < 0,001) and between *HMGA1P7* and *IGF2* expression (Spearman r = 0,7958; p < 0,001) underlines that these genes are co-regulated (Fig. 5D). Altogether, these results strongly support the idea that *HMGA1P7* could act as ceRNAs in human breast cancer and represent a novel potential mechanism accounting for *H19* and *IGF2* upregulation in these tumors.

## Discussion

lncRNAs are involved in regulating the complexity of biological processes with specific regulatory mechanisms, thereby, attracting considerable research interest^38^. We have previously isolated and characterized two pseudogenes, *HMGA1P6* and *HMGA1P7*, for the *HMGA1* gene and demonstrated that they act as decoys for *HMGA1*-targeting miRNAs^17^. In fact, their overexpression enhances HMGA1 protein levels whereas their knocking down results in the reduction of *HMGA1* mRNA and protein amounts. Moreover, their decoy activity protected the synthesis of other proteins involved in carcinogenesis^17^,^18^. In this study, we used RNA-seq technology to identify additional mRNAs differentially expressed in MEFs transgenic for *HMGA1P7*.

We found that the expression of several genes were influenced by *HMGA1P7* including also genes involved in cancer progression such as *Col6a3, Mki67, H19, Igf2, Gpx3* and *Leprel1*^30^,^31^,^32^,^33^,^34^,^35^,^36^,^37^. Indeed, oncomine analyses and tissue-microarray immunohistochemistry showed overexpression of COL6A3 in colorectal carcinomas that was significantly and directly correlated with Dukes stage, T stage, stage, recurrence and smoking status and then with a poor prognosis^30^. The MKi-67 protein (also known as Ki67) is a cellular marker for proliferation. Ki-67 protein is expressed during all active phases of the cell cycle (G_1_, S, G_2_, and mitosis), but is absent from resting cells (G_0_)^32^. *GPX3* gene codes for the Glutathione peroxidase 3, also known as plasma glutathione peroxidase (GPx-P), the variations in activity of GPX1, GPX2, and GPX3 isoforms may be associated with the development of cancers, for example, prostate cancer or even colon cancer^39^. Leprecan-like 1 is a potential tumor suppressor gene since it has been demonstrated to be downregulated in the hepatocarcinoma tissues and its overexpression inhibits cancer cell proliferation and colony formation through regulation of the cell cycle by downregulation of cyclins^40^.

Deregulation of *H19* noncoding gene was found in many tumors such as hepatocellular and bladder cancer^22^,^23^. Finally, *IGF2* overexpression is widely reported in pediatric and adult tumors^27^, and several studies involve *IGF2* as a key factor leading to cancerogenesis^27^,^28^,^29^.

Among the most deregulated genes, we selected and studied *H19* non-coding gene and *Igf2,* that share several miRNAs with *HMGA1P7*. Here, we report the ceRNA relationship between *HMGA1P7, H19* and *Igf2*. We demonstrate that *HMGA1P7* overexpression increases *H19* and *Igf2* levels inhibiting their mRNA suppression by miRNAs that target *HMGA1P7* gene, namely, miR-15, miR-16, miR-214, and miR-761. Interestingly, preliminary results show an analogous ceRNA connection between *H19, Igf2* and *HMGA1P6,* supporting the oncogenic role of the both *HMGA1* pseudogenes.

Finally, we show that expression of *HMGA1P7* significantly correlates with *H19* and *IGF2* levels in human breast cancer, suggesting the upregulation of *HMGA1P7* may increase *H19* and *IGF2* expression by a ceRNA mechanism then contributing to cancer progression. Interestingly, the oncogenic role of *HMGA1P7* is also supported by the development of malignant hematological neoplasias in *HMGA1P7* transgenic mice (manuscript in preparation).

Then, the data reported here confirm the oncogenic role of the *HMGA1P7* pseudogene that is exerted by the increased expression through a ceRNA mechanism of HMGA1 and other cancer-related genes. Future studies need, however, to characterize other genes regulated by the HMGA1 pseudogenes and thereby better define the mechanisms by which they can contribute to cancer progression.

## Materials and Methods

### Cell culture and transfections

MEFs and MCF7 were cultured in DMEM supplemented with 10% foetal calf serum (Thermo Fisher Scientific Inc). NIH3T3 cells were maintained in DMEM supplemented with 10% calf serum (Thermo Fisher Scientific Inc), glutamine and antibiotics. MycoAlert (Lonza) was regularly used to test that cells were not infected by mycoplasma. Lipofectamine plus reagent was used to transfect the cells (Thermo Fisher Scientific Inc) according to the manufacturer’s instructions. The transfected cells were selected in a medium containing geneticin (Sigma). Transfection efficiency was tested for each experiment by assessing GFP signal. To inhibit *Dicer* and *Igf2* expression, small interfering RNAs and corresponding scramble small interfering RNAs were designed and used as suggested by the manufacturer (RIBOXX).

### RNA-sequencing

RNA samples were initially checked for quality and quantity using a Bioanalyzer with the total RNA Pico chip (Agilent Technologies, Inc) and a Qubit^®^ with RNA Assay Kit (Thermo Fisher Scientific Inc) respectively.

Spike-In Mix 1 and Spike-In Mix 2, each containing the full complement of 92 polyadenylated transcripts from the ERCC plasmid reference library, were added to samples.

mRNA was selected from total RNA preparation using MicroPolyA Purist kit (Ambion, Inc).

SOLiD™ Total RNA-Seq Kit (Life Technologies Corporation) was used to convert RNA transcripts into a cDNA library, starting from low input amounts of poly(A) RNA, for analysis on the 5500 Genetic Analysis System. First of all, mRNA was incubated in a thermal cycler at 95 °C for 10 minutes to fragment the RNA by chemical hydrolysis. The RNA digested was hybridized and ligated with Solid specific adaptors. Two rounds of size selection using Agencourt^®^ AMPure^®^ XP Reagent were performed to increase the percentage of library inserts that were in the desired size range, >150 bp. The purified DNA was amplified and barcoded by 18 PCR cycles to enable sequencing of all the samples in a single multiplexed SOLiD System sequencing run.

The yield and size distribution of the amplified DNA libraries was assessed running the samples on an Agilent^®^ 2100 Bioanalyzer™ Instrument with the DNA HS Kit following the manufacturer’s instructions and Qubit^®^ dsDNA HS kit.

Ninety nanograms of each library was pooled together and one E80 emulsion was prepared following SOLiD^®^ EZ Bead™ System. About 400 millions of pooled templated beads were deposited on a 4 lanes of a 6 lanes slide and the sequencing was performed up to a read length of 50 bp, based on 5500 Genetic Analysis System Run sequencer protocol.

### Bioinformatic Analysis

Four samples were analysed: two from WT and two from *HMGA1P7* transgenic MEFs. The comparison performed was WT versus transgenic, two biological replicates for condition.

Sequencing reads in SOLiD “xsq” format were mapped against the reference genome (UCSC GRC38/mm10); reference gene structure was Refseq from the refGene.txt file of the UCSC genome browser FTP site; the mapping software was the Whole Transcriptome Analysis module from the Lifescope 2.5.1 Genomic Analysis Software analysis suite from Applied Biosystems/ThermoFisher Scientific.

A filter file, containing 6415 sequences (sequencing adaptors; barcodes; tRNAs; rRNAs; rRNA fragments; repetitive sequences; ERCC RNA sequences) was used (1) to filter the transcripts for non-significant reads and (2) to quantify the absolute expression using the External RNA Controls Consortium (ERCC) RNA Spike-In Mix.

The genome-mapped reads were then correlated with Refseq genes and the resulting gene-associated read counts were analysed with a Genomnia proprietary procedure based on the Bioconductor library edgeR^41^. The chosen limit for evaluating differential expression was 5 counts per millions in at least half of the examined samples. The normalization procedure used was the standard for edgeR (TMM). Genes were called differentially expressed when the comparison was evaluated with a FDR < 0.05. Absolute gene expression was evaluated from the read counts in RPKM (Reads per kilo base per million mapped reads).

Primary gene annotation was performed using the Bioconductor libraries biomaRt and GOstats, while functional clustering of the genes was performed using the DAVID functional annotation web site (https://david.ncifcrf.gov/).

### Mouse embryo and tissue samples

The use of mouse embryos and tissues and the experiments performed in this study were approved by the Ministero della Salute; the methods and experiments were carried out in accordance with the approved guidelines by the Ministero della Salute.

### Human breast tissue samples

Normal and neoplastic human breast tissues were obtained from surgical specimens and immediately frozen in liquid nitrogen. 32 breast samples were collected at the Institute of Pathology, University of Basel, Switzerland. The tumor samples were frozen until required for RNA extraction. The use of human tissues and the experiments performed in this study were approved by the Institute of Pathology, Molecular Pathology Division, University of Basel; the methods and experiments were carried out in accordance with the approved guidelines by the University of Basel. We declare that informed consent for the scientific use of biological material was obtained from all patients.

### RNA extraction and quantitative reverse transcription PCR

Total RNA was extracted from cells or tissues with TRIsure (Aurogene) according to the manufacturer’s instructions. For mRNA trascripts detection, we reverse transcribed total RNA from samples by using the QuantiTect Reverse Transcription Kit (Qiagen), and then Real-time PCR was performed by using Power SYBR Green PCR Master Mix (Bio-Rad) and the following primers:

*HMGA1P7*-Fw 5′-gctccttctcggctcctc-3′

*HMGA1P7*-Rev 5′-gcttgggcctcttttatgg-3′

*mIgf2* Fw 5*′-*cctccttacccaacttcaggt-3′

*mIgf2* Rv 5′-aagagatgagaagcaccaacatc-3′

*mh19* Fw 5′-atgtcttcatttctccctatagcc-3′

*mh19* Rv 5′-gtcatcctcgccttcagtg-3′

*mG6pd*-Fw5′-cagcggcaactaaactcaga-3′

*mG6pd*-Rev 5′-ttccctcaggatcccacac-3′

*mCol6a3* Fw 5′-ggaggtgtacaggaagttccac-3′

*mCol6a3* Rev 5′-gactgagccgtcaaagagga-3′

*mMki67* Fw 5′-gctgtcctcaagacaatcatca-3′

*mMki67* Rev 5′-ggcgttatcccaggagact-3′

*mGpx3* Fw 5′-gtgaacggggagaaagagc-3′

*mGpx3* Rev 5′-tgagcccaggagttctgc-3′

*mLeprel1* Fw 5′-tggaccctctttaccgagaa-3′

*mLeprel1* Rev 5′-tgatccaagatggcaatcac-3′

*hActin Fw* 5′-ccaaccgcgagaagatga-3′

*hActin Rv* 5′-ccagaggcgtacagggatag-3′

*hH19* Fw 5′-ttacttcctccacggagtcg-3′

*hH19* Rv 5′-gagctgggtagcaccatttc-3′

*hIGF2* Fw 5′-gctggcagaggagtgtcc-3′

*hIGF2* Rv 5′-gggattcccattggtgtct-3′

The 2^−∆∆CT^ formula was used to calculate the differential gene expression, and described elsewhere^42^.

### Plasmid and miRNA oligonucleotides

For transfection of miRNA oligonucleotides, cells were transfected with 50 nmol/ml of miRNA precursors or with a control no-targeting scrambled oligonucleotides (Thermo Fisher Scientific Inc) using siPORT neoFX Transfection Agent (Thermo Fisher Scientific Inc). For transfection of Anti miR-16 oligonucleotides, cells were transfected with 50 nmol/ml of Anti miR-16 or with a control no-targeting scrambled oligonucleotides (Thermo Fisher Scientific Inc).

For *Igf2* luciferase reporter construct (pGL3-*Igf2*), the miRNA seed sequence conteining fragment of *Igf2* gene (ENSMUST00000000033) was amplified by using the primers:

*Igf2* Fw 5′-aatttctagacccaaaatctcacttttccc-3′

*Igf2* Rev 5′-aatttctagagatggcccataggtgtgctc-3′.

The amplified fragment was cloned into pGL3-Control luciferase reporter vector (Promega).

All the generated vectors were confirmed by sequencing. The Renilla luciferase vector (pRL-CMV), for transient transfection efficiency, was purchased from Promega.

### Protein extraction, western blotting and antibodies

Protein extraction and Western blotting were performed as previously described^43^,^44^. The primary antibodies used were anti-IGF2 (#32592) from Sabbiotech; anti-GAPDH (sc-32233) and anti-γ-Tubulin (sc-17787) from Santa Cruz Biotechnology. Blots were visualized by using the Western blotting detection reagents (Thermo Fisher Scientific Inc).

### Dual-luciferase reporter assay

For dual-luciferase reporter assay, 3 × 10^5^ NIH3T3 cells were co-transfected in 6-well plates with the pGL3- *Igf2* or the pGL3-*H19* luciferase reporter vectors, together with the Renilla luciferase plasmid and miRNA precursors or a control no-targeting scrambled oligonucleotides (Thermo Fisher Scientific Inc), using siPORT neoFX Transfection Agent (Thermo Fisher Scientific Inc). The pRL-TK control vector expressing Renilla luciferase (Promega) was used for normalization of cell number and transfection efficiency. Luciferase activity was measured 48 hours after transfection using the Dual-Luciferase Reporter Assay System (Promega) with a Lumat LB 9507 apparatus (Berthold Technologies).

### Growth curve assay

For each experimental point 3 × 10^4^ cells were plated in a 60 mm plate. Cells were counted in triplicate for 5 days with Burker hemocytometer chamber.

### Statistical analysis

Data were analyzed using a two-sided unpaired t test (GraphPad Prism, GraphPad Software, Inc.). Values of P < 0.05 were considered statistically significant. Regression analysis, correlation coefficients and statistical analysis were generated using GraphPad Prism, GraphPad Software, Inc.

## Additional Information

**How to cite this article**: De Martino, M. *et al. HMGA1P7*-pseudogene regulates *H19* and *Igf2* expression by a competitive endogenous RNA mechanism. *Sci. Rep.* **6**, 37622; doi: 10.1038/srep37622 (2016).

**Publisher’s note:** Springer Nature remains neutral with regard to jurisdictional claims in published maps and institutional affiliations.
